# Use of a Force-Torque Sensor for Self-Calibration of a 6-DOF Medical Robot

**DOI:** 10.3390/s16060798

**Published:** 2016-05-31

**Authors:** Ahmed Joubair, Long Fei Zhao, Pascal Bigras, Ilian A. Bonev

**Affiliations:** École de Technologie Supérieure (ÉTS), Montreal, QC H3C 1K3, Canada; longfei.zhao.1@ens.etsmtl.ca (L.F.Z.); pascal.bigras@etsmtl.ca (P.B.); ilian.bonev@etsmtl.ca (I.A.B.)

**Keywords:** robot calibration, robot accuracy, observability, medical robot, robot kinematic, robotic metrology

## Abstract

The aim of this paper is to improve the position accuracy of a six degree of freedom medical robot. The improvement in accuracy is achieved without the use of any external measurement device. Instead, this work presents a novel calibration approach based on using an embedded force-torque sensor to identify the robot’s kinematic parameters and thereby enhance the positioning accuracy. A simulation study demonstrated that our calibration approach is effective, whether or not any measurement noise is present: the position error is improved, inside the robot target workspace, from 12 mm to 0.320 mm, for the maximum values, and from 9 mm to 0.2771 mm, for the mean errors.

## 1. Introduction

Medical robots show a promising future in various health issues in the most recent decades. With recent developments in sensors and control theory, medical robots provide many inspiring solutions in the fields of: diagnosis, surgery, orthopedics, rehabilitation, prosthetics and exoskeletons, *etc.* [1,2]. The force-torque (wrench) sensor is an essential component of these medical robot applications. MedRUE [3], OTELO [4] and Hippocrate [5] robot systems were developed for the ultrasound scanning of vascular diseases. In all three robot systems, force-torque sensors are employed to maintain proper contact with the patient’s body during the examination. The Black Falcon system, which is a fundamental study for many other surgical robot systems, allows the surgeon to feel the interaction with tissue and thereafter improve the surgical performance. The Da Vinci system is a popular surgery robot widely used in hospitals [6]. The adoption of force-torque sensors in Da Vinci system has been studied in depth [7]. The Robodoc assistant system is a medical orthopedic robot for use during total knee replacement, and it achieves results that are comparable to technician performance [8]. The force-torque sensors are used in Robodoc for both control and safety reasons.

Medical robots have also contributed to the field of rehabilitation. The InMotion ARM, which is based on the MIT-Manus project, is an interactive robotic system for upper-extremity rehabilitation therapy [9,10], and the robotic stepper is a device, developed by the National Aeronautics and Space Administration (NASA), to help patients with lower-extremity rehabilitation [11]. Force-torque sensors are employed in these rehabilitation robots to measure the strength and the capability of the patient. Medical robots have also been developed as substitutes for malfunctioning parts of the human body. The I-limb ultrasound system and the ReWalk system are exoskeleton robots for hand prosthetics and leg prosthetics, respectively [12,13]. Force-torque sensors are used in the prosthetic and exoskeleton robots to control the joints and to evaluate the power of the limb movements.

Medical robots often need to be accurate, not just repeatable, which means that they must be calibrated. Most robot calibration approaches are based on minimizing the pose residual, which involves external measurement devices such as a coordinate measurement machine (CMM) [14,15], laser tracker [16,17,18], measurement articulated arm [14], ball-bar [19,20], or a high-accuracy touch probe [21,22]. However, these measurement devices tend to be expensive, and they are not readily available. Furthermore, even though other studies have developed low-cost calibration methods, such as in [19,20], they still require the use of external measurement devices.

Other robotic applications are dedicated to measuring and/or reproducing human movements, such as [23], which presents a methodology to accurately record human finger postures during grasping. In this work, human finger postures are measured during grasping. As with the aforementioned works, measurements are taken with external measurement devices: an optical tracking of markers that are attached to the skin of the hand, and tracked using stereo-cameras. The considered kinematic parameters in this work are geometric static parameters, and parameters controlling the location of the bones and the joint markers. These parameters are identified by using a constrained least-squares minimization. The minimization problem is solved by employing a primal-dual interior point. It minimizes the residuals of the coordinates of measured markers and the corresponding estimated coordinates, which are a function of the static parameters and joint angle values.

Force-torque sensors are already used in many medical robot systems, so it makes sense to use these sensors, rather than external coordinate measurement equipment, for calibrating the robots (*i.e.*, improving the robot positioning accuracy). Yet, to the best of our knowledge, no such calibration methods have been proposed in the literature. In previous work, other measurement approaches were used, such as Cartesian coordinates [14,15,16,17,18], or distance measurements [19,20]. The novelty of our work is the use of a force-torque sensor to improve the positioning accuracy of a medical robot (MedRUE). The robot parameters are identified by minimizing the force and torque residuals, instead of minimizing the residuals of the end-effector position and/or orientation, as done in conventional approaches [14,15,16,17,18,19,20]. The sensor we used is the embedded force-torque sensor, located between the flange and the tool. Thus, our calibration approach can be considered as a self-calibration method.

The proposed approach could be used for any other medical or industrial robot. Industrial robots are not always equipped with force-torque sensors. However, such sensors are readily available in the market and can be easily installed. Indeed, industrial serial robots are increasingly using these sensors for programming purposes: the force-torque sensors are installed in order to move manually the robot end-effector, during the online programming (also called lead-through programming).

In the identification process proposed in this paper, the data are collected by the robot’s force-torque sensor. The process of identifying the parameters is based on minimizing the residual of the force and torque at the robot’s end-effector. The accurate identification of the robot’s parameters leads to improved position accuracy. Our approach is validated through a simulation, in which the position accuracy is evaluated before and after calibration.

This paper is organized as follows. The next section describes the force and torque forward kinematic equations, followed by a description of the calibration approach. We then present our simulation study, followed by a results analysis. Finally, we discuss our conclusions and suggestions for further work in the last section of the paper.

## 2. Robot Description and Forward Kinematics

### 2.1. Robot Description and the Main Reference Frames

**Figure 1:**
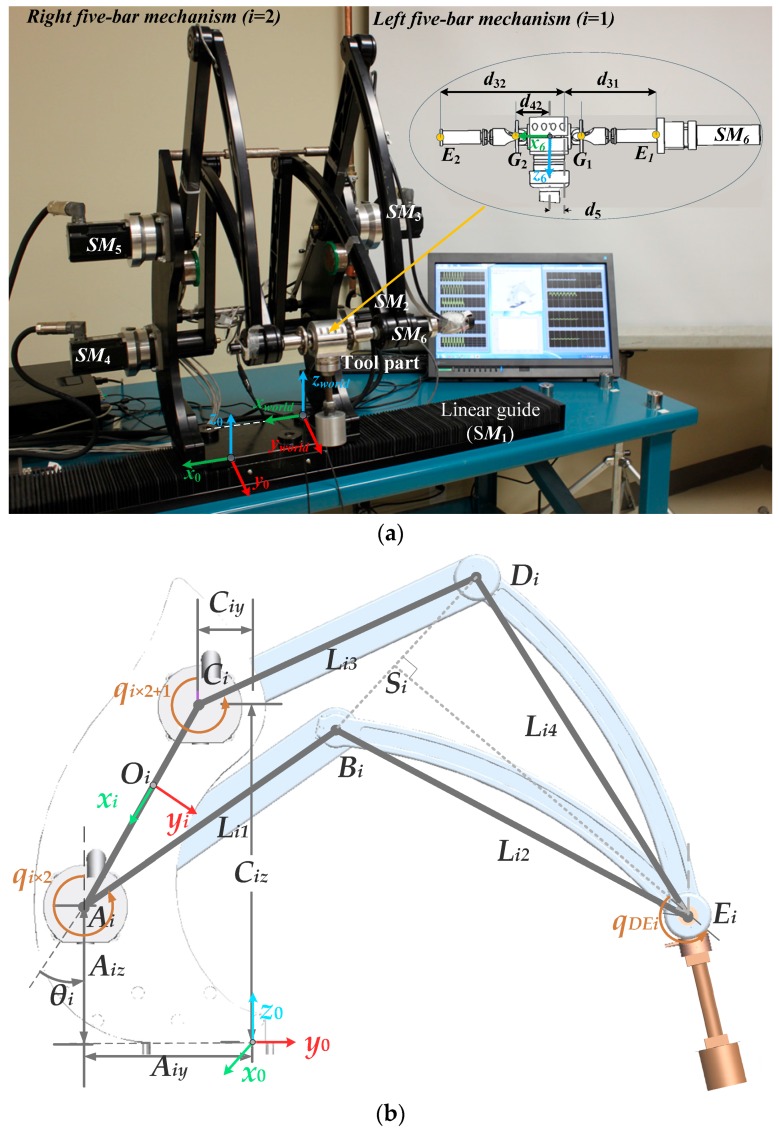
(**a**) The MedRUE robot prototype with the tool part and (**b**) a five-bar mechanism of the MedRUE where (*i* = 1, 2).

The MedRUE robot (Figure 1a) is a medical robot dedicated for vascular ultrasound examination. MedRUE is a six degrees of freedom (6-DOF) hybrid serial-parallel robot. It is composed of two five-bar mechanisms (Figure 1b), which are symmetrically assembled. These mechanisms are considered to be perfectly parallel to each other and perpendicular to the robot base. The robot base is fixed to a linear guide actuated through a servomotor *SM*_1_; the corresponding joint variable is denoted by *q*_1_. Five other servomotors are also used in order to actuate the robot revolute joints: *SM*_2_ and *SM*_3_, for the left five bar mechanism, and *SM*_4_ and *SM*_5_ for the right side (Figure 1a). The sixth servomotor—attached to the link having $L_{14}$ as its length—is used to rotate the probe about the *x* axis of the last reference frame (*F*_6_), which is defined as follows: the origin of *F*_6_ is located midway between *G*_1_ and *G*_2_; its *x* axis (*x*_6_) is defined to pass through *G*_1_ and *G*_2_, and *z*_6_ is pointing toward the probe center.

Each five-bar mechanism *i* (*i* = 1, 2) has five links: the distance *d_i_* between the anchor points of the two proximal links, and the four mobile links having *L_ij_* (*j* = 1, 2, 3, 4) as lengths. The five links connect five revolute joints (*A_i_, B_i_, C_i_, D_i_, E_i_*), among which only two (*A_i_* and *C_i_*) are actuated through servomotors *SM_i_*_×2_ and *SM_i_*_×2+1_: the corresponding two angles are denoted *q_i_*_×2_ and *q_i_*_×2+1_, respectively. A total of five angles of active joints are considered (*q*_2_, …, *q*_6_).

Finally, joints *E*_1_ and *E*_2_ are linked through the probe support (Figure 1a), which has a universal joint at each extremity *G_i_*. The *x* coordinate of *G_i_* with respect to the base frame is denoted by $d_{4i}$.

**Figure 2:**
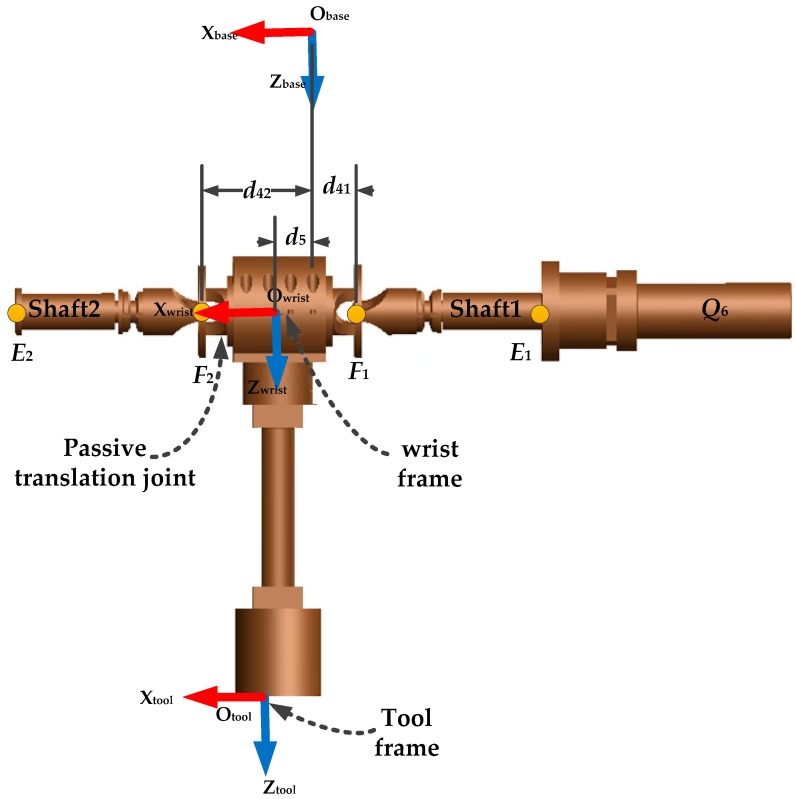
Tool part of MedRUE.

In our calibration process, nine reference frames are considered:

- *F*_0_: The reference frame of the robot base, located on the robot base at *O*_0_. As shown in Figure 1a, the *x* axis (*x*_0_) is aligned with the axis of the linear guide, and *z*_0_ is normal to the plane defined by the platform of the robot base. The translation $T_{0}^{world}={\left[\begin{array}{ccc} x_{0} & y_{0} & z_{0} \end{array}\right]}^{T}$ and the orientation (*α*_0_, *β*_0_, *γ*_0_), described in XYZ fixed Euler angles, of *F*_0_ with respect to *F_world_*, are expected to be identified by the calibration process.
- *F_world_*: The world reference frame (Figure 1a), associated with the robot work-cell. It has approximately the same orientation as *F*_0_.
- *F*_1_ to *F*_6_: The reference frames associated with the joints (*F*_1_, *F*_2_, …, *F*_6_). These frames are not shown.
- *F_tool_*: The tool reference frame associated with the robot probe (Figure 2). The origin of *F_tool_* is described to be the center of the end-effector (*i.e.*, the probe), and its orientation is considered to be the same as that of *F*_6_. Knowing that the end-effector orientation is not used in our calibration process, therefore, only the translation $T_{\text{tool}}^{6}={\left[\begin{array}{ccc} x_{t} & y_{t} & z_{t} \end{array}\right]}^{T}$ of *F*_tool_ with respect to *F*_6_ is considered.

### 2.2. Position Equations

Given the vector **ψ** = [*q*_1_, *q*_2_, …, *q*_6_]^T^ of the active joint variables, the end-effector’s pose with respect to the world frame is represented by homogeneous matrices as follows:

(1) $$A_{\text{tool}}^{\text{world}}(\psi )=A_{\text{base}}^{\text{world}}A_{\text{wrist}}^{0}A_{\text{sensor}}^{\text{wrist}}A_{\text{tool}}^{\text{sensor}}$$

where $A_{a}^{b}$ denotes the homogeneous matrix representing a frame *a* with respect to a frame *b*, and can be represented in the matrix form of $\left[\begin{array}{cc} R_{a}^{b} & T_{a}^{b}\\ 0 & 1 \end{array}\right]$.

The rotation matrix is often represented by **R**(*α*,*β*,*γ*) = **R***_x_*(*γ*)**R***_y_*(*β*)**R***_z_*(*α*), and the translation matrix by **T**(*x*,*y*,*z*) = **T***_x_*(*x*)**T***_y_*(*y*)**T***_z_*(*z*), where **R***_u_*(*ϕ*) and **T***_u_*(*d*) are the rotation/translation operator along the *u* axis with value *ϕ*/*d*. Since robot joints are only included in $A_{\text{wrist}}^{0}$, then $A_{\text{base}}^{\text{world}}$, $A_{\text{sensor}}^{\text{wrist}}$ and $A_{\text{tool}}^{\text{sensor}}$ in Equation (1) are constant matrices, which can be defined directly by parameter sets [*x*_0_, *y*_0_, *z*_0_*, α*_0_, *β*_0_, *γ*_0_], [*x*_S_, *y*_S_, *z*_S_, *α*_S_, *β*_S_, *γ*_S_] and [*x*_T_, *y*_T_, *z*_T_, *α*_T_, *β*_T_, *γ*_T_]. The following paragraphs present the calculation of $A_{\text{wrist}}^{0}$.

The coordinates of $B_{i}$ and $D_{i}$ are expressed with respect to the local frame F*_i_* on *i*th five-bar mechanism as follows:

(2) $$r_{O_{i}B_{i}}=r_{O_{i}A_{i}}+{\left[\begin{array}{ccc} L_{i1}\cos\left(q_{i\times 2}+\delta q_{i\times 2}\right) & L_{i1}\sin\left(q_{i\times 2}+\delta q_{i\times 2}\right) & 0 \end{array}\right]}^{T}$$

(3) $$r_{O_{i}D_{i}}=r_{O_{i}C_{i}}+{\left[\begin{array}{ccc} L_{i3}\cos\left(q_{i\times 2+1}+\delta q_{i\times 2+1}\right) & L_{i3}\sin\left(q_{i\times 2+1}+\delta q_{i\times 2+1}\right) & 0 \end{array}\right]}^{T}$$

where *L_i_*_1_ and *L_i_*_3_ are the lengths of the four swinging links as shown in Figure 1, and $\delta q_{i}$ is the offset of *i*th active joint. The vector $r_{O_{i}A_{i}}$ is calculated as follows:

(4) $$r_{O_{i}A_{i}}={\left[\begin{array}{ccc} \frac{\sqrt{{\left(C_{iy}-A_{iy}\right)}^{2}+{\left(C_{iz}-A_{iz}\right)}^{2}}}{2} & 0 & 0 \end{array}\right]}^{T}$$

and

(5) $$r_{O_{i}C_{i}}=-r_{O_{i}A_{i}}$$

The coordinates of $E_{i}$ with respect to a frame F*_i_* are obtained as follows:

(6) $$r_{O_{i}E_{i}}=r_{O_{i}D_{i}}+r_{D_{i}S_{i}}+r_{S_{i}E_{i}}$$

where

(7) $$r_{D_{i}S_{i}}=\frac{r_{D_{i}B_{i}}}{2}\left(\frac{L_{i4}^{2}-L_{i2}^{2}}{{\left\|r_{D_{i}B_{i}}\right\|}^{2}}+1\right)$$

(8) $$r_{S_{i}E_{i}}=\sqrt{L_{i4}^{2}-{\left\|r_{D_{i}S_{i}}\right\|}^{2}}R_{z}\left(\frac{\pi }{2}\right){\hat{r}}_{D_{i}B_{i}}$$

and ${\hat{r}}_{D_{i}B_{i}}$ is the unit vector along $r_{D_{i}B_{i}}=r_{O_{i}B_{i}}-r_{O_{i}D_{i}}$.

The coordinates of $E_{i}$ w.r.t. F_base_ are obtained by a transformation matrix $A_{i}^{0}$ as follows:

(9) $$r_{O_{0}E_{i}}=A_{i}^{0}r_{O_{i}E_{i}}$$

where

(10) $$A_{i}^{0}=\left[\begin{array}{cccc} 0 & 0 & 1 & q_{1}+{\left(-1\right)}^{i}d_{4i}\\ -\sin\left(\theta_{i}\right) & \cos\left(\theta_{i}\right) & 0 & \frac{A_{iy}+C_{iy}}{2}\\ -\cos\left(\theta_{i}\right) & -\sin\left(\theta_{i}\right) & 0 & \frac{A_{iz}+C_{iz}}{2}\\ 0 & 0 & 0 & 1 \end{array}\right]$$

and *θ_i_*, which is the angle between $r_{A_{i}C_{i}}$ and the normal of the *x*_0_*y*_0_ plane, is calculated as follows:

(11) $$\theta_{i}=\text{atan}2\left(y_{Ai}-y_{Ci},z_{Ci}-z_{Ai}\right)$$

The orientation of F_wrist_ w.r.t. F_base_ is obtained from the corresponding rotation matrix $R_{\text{wrist}}^{\text{base}}\left(\alpha ,\beta ,\gamma \right)$, where *α*, *β* and *γ* are the fixed XYZ Euler angles. The rotation angle along *x*_0_ is directly obtained as $\gamma =q_{DE_{1}}+q_{6}+\delta q_{6}$. According to the design of the tool part shown in Figure 2,

(12) $$R_{\text{wrist}}^{0}\left(\alpha ,\beta ,\gamma \right)\left[\begin{array}{c} 1\\ 0\\ 0 \end{array}\right]=\frac{r_{O_{0}F_{2}}-r_{O_{0}F_{1}}}{\left\|r_{O_{0}F_{2}}-r_{O_{0}F_{1}}\right\|}=\left[\begin{array}{c} u_{x}\\ u_{y}\\ u_{z} \end{array}\right]$$

Then *α* and *β* are obtained as:

(13) $$\alpha =\sin^{-1}\left(u_{y}\cos\gamma +u_{z}\sin\gamma \right)$$

(14) $$\beta =\text{atan}2\left(\begin{array}{cc} \Psi & \sqrt{{\left(\cos\alpha \right)}^{2}-\Psi^{2}} \end{array}\right)$$

where, $\Psi =u_{y}\sin\gamma -u_{z}\cos\gamma$.

The translation of F_wrist_ w.r.t. F_base_ is calculated as follows:

(15) $$T_{\text{wrist}}^{0}=r_{O_{0}F_{1}}+R_{\text{wrist}}^{0}\left[\begin{array}{c} d_{41}+d_{5}\\ 0\\ 0 \end{array}\right]$$

Finally, the pose of F_wrist_ w.r.t. F_base_ is expressed as follows:

(16) $$A_{\text{wrist}}^{0}=\left[\begin{array}{cc} R_{\text{wrist}}^{0} & T_{\text{wrist}}^{0}\\ 0 & 1 \end{array}\right]$$

### 2.3. Force and Torque Equations

**Figure 3:**
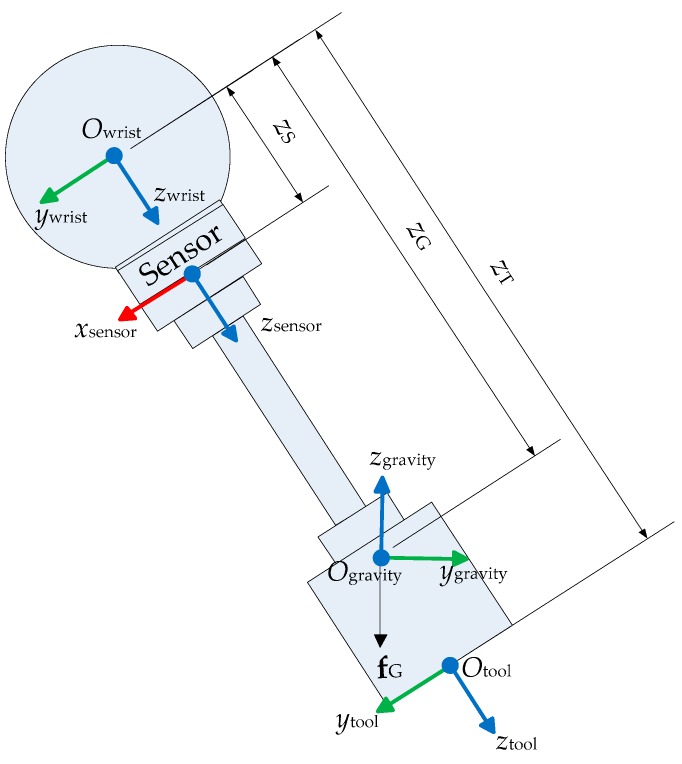
Gravity effect of the tool part on the force sensor.

The gravity frame F_gravity_ is assigned at the tool part’s center of gravity, as shown in Figure 3. When the robot is not in contact with its environment, the gravity force **f**_G_ in frame F_gravity_ is the cause of the force and torque on the force sensor. The forward kinematic solution to obtain the force and torque in force sensors is:

(17) $$F_{G}^{\text{sensor}}=A_{\text{wrist}}^{\text{sensor}}A_{\text{gravity}}^{\text{wrist}}F_{G}^{\text{gravity}}$$

where F is a 6 × 1 wrench vector composed of force and torque, and A is a 6 × 6 transformation matrix between screws. The orientation of F_gravity_ is in alignment with F_world_, rather than fixed relative to the tool part. The wrench of the gravity force of the tool part w.r.t. the F_gravity_ is

(18) $$F_{G}^{\text{gravity}}=\left[\begin{array}{l} f_{G}^{\text{gravity}}\\ 0 \end{array}\right]$$

where $f_{G}^{\text{gravity}}={\left[\begin{array}{ccc} 0 & 0 & -m_{\text{Tool}}g \end{array}\right]}^{T}$ with *m*_Tool_ is the mass of the tool part and *g* is the gravitational constant. Since gravity is a pure force, $\tau_{G}^{\text{gravity}}={\left[\begin{array}{ccc} 0 & 0 & 0 \end{array}\right]}^{T}$.

The transformation matrix $A_{\text{gravity}}^{\text{writst}}$ can be expressed as

(19) $$A_{\text{gravity}}^{\text{writst}}=\left[\begin{array}{cc} R_{\text{gravity}}^{\text{writst}} & 0\\ p_{\text{gravity}}^{\text{writst}}\times R_{\text{gravity}}^{\text{writst}} & R_{\text{gravity}}^{\text{writst}} \end{array}\right]$$

Since F_gravity_ keeps the same orientation with F_world_, then $R_{\text{gravity}}^{\text{wrist}}=R_{\text{world}}^{\text{wrist}}={\left(R_{\text{base}}^{\text{world}}R_{\text{wrist}}^{\text{base}}\right)}^{T}$. $p_{\text{gravity}}^{\text{wrist}}={\left[\begin{array}{ccc} x_{G} & y_{G} & z_{G} \end{array}\right]}^{T}$ is the translation offset of the origin of F_gravity_ w.r.t. F_wrist_. $p_{\text{gravity}}^{\text{wrist}}\times$ is the vector product operation, and it is equal to

(20) $$p_{\text{gravity}}^{\text{wrist}}\times =\left[\begin{array}{ccc} 0 & -z_{G} & y_{G}\\ z_{G} & 0 & -x_{G}\\ -y_{G} & x_{G} & 0 \end{array}\right]$$

Similar to Equation (19), $A_{\text{wrist}}^{\text{sensor}}$ is characterized by parameters describing F_sensor_ w.r.t. F_wrist_:

(21) $$A_{\text{wrist}}^{\text{sensor}}={\left(A_{\text{sensor}}^{\text{wrist}}\right)}^{-1}=\left[\begin{array}{cc} {\left(R_{\text{sensor}}^{\text{wrist}}\right)}^{T} & 0\\ -{\left(R_{\text{sensor}}^{\text{wrist}}\right)}^{T}p_{\text{sensor}}^{\text{wrist}}\times & {\left(R_{\text{sensor}}^{\text{wrist}}\right)}^{T} \end{array}\right]$$

The sensor reference frame F_sensor_ is defined in the information given by the sensor manufacturer. During assembly, its orientation w.r.t. F_wrist_ is expressed by Euler-XYZ angles:

(22) $$R_{\text{sensor}}^{\text{wrist}}=R_{x}\left(\gamma_{S}\right)R_{y}\left(\beta_{S}\right)R_{z}\left(\alpha_{S}\right)$$

Similar to Equation (20), $p_{\text{sensor}}^{\text{wrist}}\times$ is the vector product operation of $p_{\text{sensor}}^{\text{wrist}}={\left[\begin{array}{ccc} x_{S} & y_{S} & z_{S} \end{array}\right]}^{T}$.

## 3. Parameters Used During Calibration

In our robot calibration process, the following parameters were considered:

- The lengths of the ten links of the two five-bar mechanisms: *L*_11_, *L*_12_, *L*_13_, *L*_14_, *L*_21_, *L*_22_, *L*_23_ and *L*_24_.
- The *y* and *z* coordinates of the anchor points of the two proximal links of the five-bar mechanisms: *A*_1*y*_, *A*_1z_, *C*_1*y*_, *C*_1*z*_, *A*_2*y*_, *A*_2z_, *C*_2*y*_, *C*_2*z*_.
- The offsets of the six active joints: *δq*_1_, *δq*_2_, *δq*_3_, *δq*_4_, *δq*_5_, *δq*_6_.
- The offset parameters for the tool part: *d*_31_, *d*_32_, *d*_41_, *d*_42_, *d*_5_.
- The parameters defining the base with respect to the world frame: *x*_0_, *y*_0_, *z*_0_, *α*_0_, *β*_0_, *γ*_0_.
- The position of the tool frame with respect to the wrist frame: *x*_T_, *y*_T_, *z*_T_, *α*_T_, *β*_T_, *γ*_T_.
- The parameters to describe the sensor frame w.r.t. the wrist frame: *x*_S_, *y*_S_, *z*_S_, *α*_S_, *β*_S_, *γ*_S_.
- The parameters to describe the offset of gravity frame w.r.t. the wrist frame: *x*_G_, *y*_G_, *z*_G_.
- The mass of the tool part: *m*_Tool_.

Of the 46 parameters that we considerd, a total of 17 parameters are non-identifiable, which means that we need to reduce the number of identifiable parameters to 29.

## 4. Calibration Process

Our calibration process is explained in detail in Section 4.1, Section 4.2 and Section 4.3. Its main steps are presented in what follows:

1. Develop the calibration model: the forward kinematics, presented in Section 2.2.
2. Create a pool **Ω** of 40,000 configurations uniformly distributed inside the whole robot workspace. Create a set **Ω**_t_ of 336 configurations uniformly distributed inside the target workspace (see Section 4.3). We note that the configurations of the set **Ω**_t_ are different from these of **Ω**.
3. Select 100 configurations to be used in the identification process. These configurations are chosen through an observability analysis, as explained in Section 4.1.
4. Take the force and torque measurements, for all robot configurations (**Ω**_t_ and **Ω**). Measurements are done by using the robot force-torque sensor. We note that in this paper all measurements are generated by simulation, as explained in Section 4.3 and Section 5.
5. Identify the robot parameter values by using the calibration configurations selected in step 3; the identification approach is presented in details in Section 4.2.
6. Evaluate the accuracy after calibration, as explained in Section 5.

### 4.1. Selection of Calibration Configurations

After creating the calibration model, and generating a pool **Ω** of 40,000 configurations uniformly distributed inside the whole robot workspace, a set of 100 calibration configurations is selected among **Ω**. This is done by using an approach commonly called *observability analysis*. This analysis is used to obtain the optimal set of the calibration configurations, and is based on the singular value decomposition (SVD) of the *identification Jacobian matrix* **J**. The matrix **J** is composed of the derivatives of the end-effector force and torque vector (Equation (17)), with respect to all of the robot independent parameters. The Jacobian matrix is also used in the linearization of the force and torque equations (Equation (17)), around the calibration configurations (*i.e.*, Taylor approximation). This linearization allows identifying the parameter values, as explained in Section 4.2. The nominal values of the robot’s independent parameters are represented by the vector **p_nom_**. The matrix **J** is calculated as follows, for *i* = 1…*n*.

(23) $$J=\left[\begin{array}{c} J_{1}\\ \vdots \\ J_{n} \end{array}\right]$$

where J*_i_* is the 6 × *m* Jacobian matrix at the *i*th calibration configuration, *n* is the number of calibration configurations, and *m* is the number of considered parameters (not all of which are necessarily identifiable). In our case, *n* = 100 and *m* = 49.

**J**_i_ is given by:

(24) $$J_{i}=\left[\begin{array}{c} \begin{array}{c} \frac{\partial F_{x,i}}{\partial p_{\text{nom}}}\\ \frac{\partial F_{y,i}}{\partial p_{\text{nom}}} \end{array}\\ \begin{array}{c} \frac{\partial F_{z,i}}{\partial p_{\text{nom}}}\\ \frac{\partial T_{x,i}}{\partial p_{\text{nom}}} \end{array}\\ \begin{array}{c} \frac{\partial T_{y,i}}{\partial p_{\text{nom}}}\\ \frac{\partial T_{z,i}}{\partial p_{\text{nom}}} \end{array} \end{array}\right]$$

The matrix **J** is also used to find the non-identifiable parameters. The rank *r_J_* of the Jacobian **J** represents the number of identifiable parameters. If *r_J_* < *m*, then *m* − *r_J_* parameters are non-identifiable; the corresponding columns should be removed from **J**. This procedure is carried out using an algorithm that is based on the approach proposed in [21]. The stop criteria is when *m* becomes equal to *r_J_*. The algorithm proceeds as follows:

1. Remove all zero columns from **J**. The corresponding parameters have no impact on the calibration model.
2. Calculate the condition number, c_J_, of J. The condition number is used to evaluate how good is the matrix **J** for the parameter identification. With a bad condition number (high value), the solutions are unstable with respect to small changes in measurement errors. Therefore, to have a robust identification system, the condition number should be as small as possible.
3. Remove, one at a time, the column related to each parameter from **J**, and calculate both the new rank and condition number ($r_{J}^{*}$ and $c_{J}^{*}$) for the new Jacobian matrix J^*^. The column that, if eliminated, results in the maximum reduction of the condition number and gives the same rank ($r_{J}^{*}$ = *r_J_*), is definitively removed (*i.e.*, the corresponding parameter will be not subject to the identification process).
4. Replace **J** with J*, and repeat the process from step (2).

As a result, of the 49 parameters considered in our calibration model, 22 are non-identifiable and are indicated by the symbol ‘*’ in Table 1.

The fact that some parameters are non-identifiable is mainly due to redundancy, or the fact that they have no impact on the force and torque equations that represent the calibration model.

The parameter identification is achieved by minimizing the residual of the end-effector force and torque, which are measured by the robot’s force sensor (Figure 3); no external measurement device is required. Further, only the gravity effect of the end-effector is used to apply forces to the robot’s end-effector. Therefore, to change the applied force on the end-effector, it is necessary to change its orientation.

In our identification process, 100 calibration configurations are selected from among the 40,000 configurations. The remaining 39,900 configurations are used for validation purpose. The 40,000 configurations are uniformly distributed on three layers, within the entire robot workspace. Note that several orientations are generated for each position. The calibration configurations are selected using an observability analysis, which allows us to identify the most appropriate configurations and thus identify the most effective parameters. This analysis is based on using the first observability index, denoted by *O*_1_ and calculated by using the singular value of the Jacobian identification matrix (*i.e.*, the sensitivity matrix). The procedure of selecting the calibration configurations is based on the DETMAX algorithm, which was initially proposed in [24].

**Figure 4:**
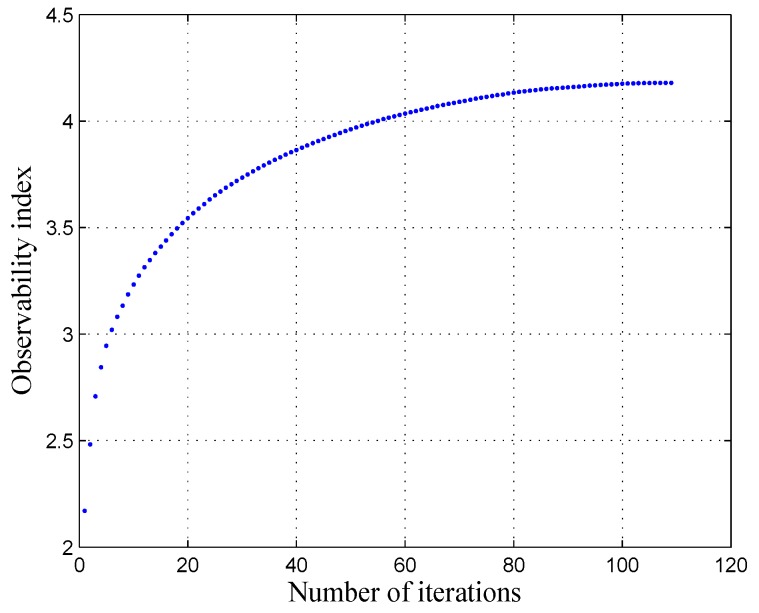
Evolution of the observability index *O*_1_ with respect to the selection algorithm iterations.

According to [25,26], the index *O*_1_ seems to be the most appropriate index for the kinematic calibration. This was also confirmed by our simulation, through a comparison of the five observability indices that were presented in the literature and thoroughly detailed in [26]. The convergence of *O*_1_ is represented in Figure 4, and is calculated as follows:

(25) $$O_{1}=\frac{{\left(\sigma_{1}\sigma_{2}\ldots \sigma_{m}\right)}^{\frac{1}{m}}}{\sqrt{n}}$$

where *n* is the number of calibration configurations, *σ*_1_ … *σ_m_* are the singular values of the Jacobian identification matrix for the *m* = 29 identifiable parameters.

### 4.2. Parameter Identification Process

The parameter identification process is based on using the Jacobian matrix **J**, which relates the force and torque errors to the 29 unknown parameter values. The matrix **J** is built by the linearization of the forward kinematics model (Equation (17)) around each calibration configuration. The parameter values are identified by means of an iterative algorithm, in which the parameters’ vector is initialized by **p_nom_**, and is updated at each iteration (*i.e.*, replaced by the vector **p**_identified_ of the identified values). The matrix **J** is also iteratively updated, since its calculation is based on **p**.

**Figure 5:**
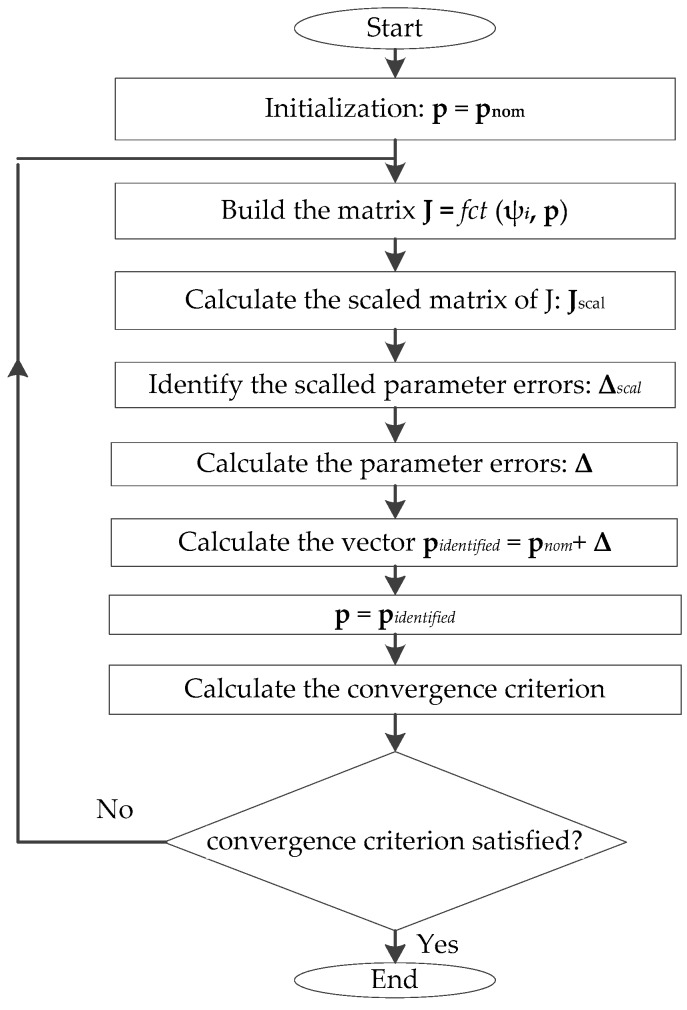
Flow chart of the parameter identification algorithm.

The identification algorithm is presented in Figure 5 and has the following steps:

(a) Matrix **J** is calculated, as explained in Section 4.1. This calculation involves **p** and the values of the vector **ψ*_i_*** = [*q*_1_, *q*_2_,…, *q*_6_] ^T^ (*i* = 1,…, *n*) of active joints of the 100 calibration configurations.
(b) A system of linear equations is formed by the measured force and torque errors, the unknown robot’s parameter errors, and the Jacobian matrix **J**. In order to maintain acceptable variance of each parameter (*i.e.*, proper convergence in the linear system), parameter scaling is implemented, by using the column scaling approach proposed in [24]. The scaled matrix obtained is denoted by **J**_scal_, and it is used to identify the robot’s scaled parameter errors (**Δ***_scal_*), as follows:
  (26) $$\Delta_{scal}={\left(J_{scal}^{T}J_{scal}\right)}^{-1}J_{scal}^{T}\;\left[\begin{array}{c} Fx_{meas,1}-Fx_{est,1}\\ \begin{array}{l} Fy_{meas,1}-Fy_{est,1}\\ Fz_{meas,1}-Fz_{est,1}\\ Tx_{meas,1}-Tx_{est,1}\\ Ty_{meas,1}-Ty_{est,1}\\ Tz_{meas,1}-Tz_{est,1} \end{array}\\ \vdots \\ Fx_{meas,n}-Fx_{est,n}\\ \begin{array}{l} Fy_{meas,n}-Fy_{est,n}\\ Fz_{meas,n}-Fz_{est,n}\\ Tx_{meas,n}-Tx_{est,n}\\ Ty_{meas,n}-Ty_{est,n}\\ Tz_{meas,n}-Tz_{est,n} \end{array} \end{array}\right]$$
  where, [*Fx_meas,i_, Fy_meas,i_, Fz_meas,i_, Tx_meas,i_, Ty_meas,i_, Tz_meas,i_*]^T^ is the vector of the measured force and torque *i*, and [*Fx_est,i_, Fy_est,i_, Fz_est,i_, Tx_est,i_, Ty_est,i_, Tz_est,i_*]^T^ is the corresponding estimated vector. The estimated vector is calculated by substituting in the forward kinematic equation (Equation (17)): The vector **p** of the parameters’ values and the vector **ψ*_i_*** of the active joint variables. The vector **p** is initialized by its nominal values **p*_nom_***, and updated after each iteration of this identification algorithm.
(c) The parameter errors, which represent the difference between the real values and the nominal values of the parameters, are denoted by **Δ**, and calculated as follows:
  (27) $$\Delta =diag{\left(D_{1},D_{2},\cdots ,D_{m}\right)}^{-1}\Delta_{scal}$$
  where *D_j_*, (*j* = 1, 2, *…*, *m*) are the scaling coefficients, defined as follows: $D_{j}=\sqrt{\sum_{i=1}^{6n}J_{i,j}^{2}}$. Also, *n* is the number of calibration configurations, and **J***_ij_* is the element of the Jacobian matrix located at the *i*th row and the *j*th column.
(d) Finally, the vector of the identified parameter values is
  (28) $$p_{identified}=p_{\text{nom}}+\Delta$$

To converge towards a solution for the unknown parameter values, an iterative Newton-based procedure was used. After **p**_identified_ has been calculated, the **p** vector is replaced by the last **p**_identified_ vector obtained, and the estimation process is restarted from step (a).

Steps (a) to (d) are repeated until reaching a convergence criterion, which is the root mean square error (*RMSE*) between two successive iterations. The *RMSE* is evaluated between the vector of the latest identified parameters and the previous one. The convergence criterion was set to 10^−16^, and the system converged towards a solution after five iterations.

### 4.3. Validation after Calibration

**Figure 6:**
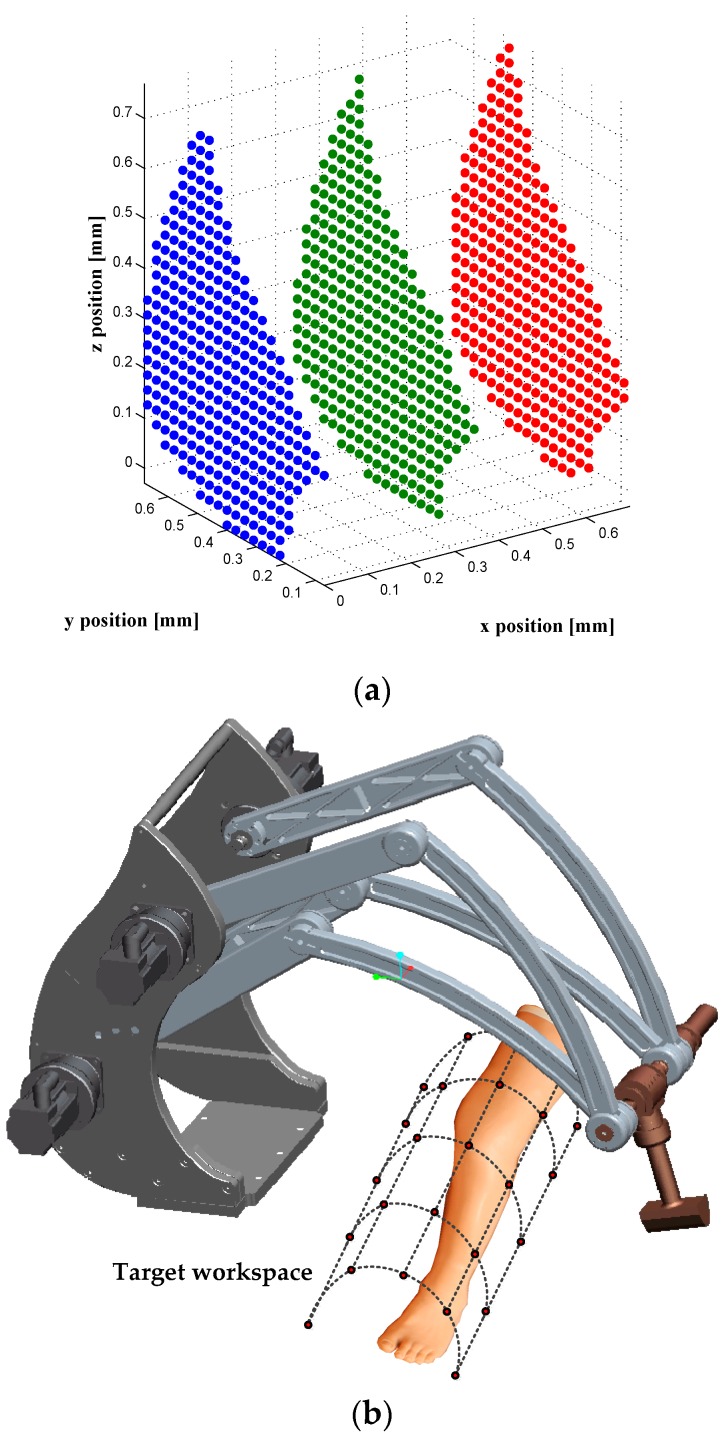
The positioning of the robot end-effector within (**a**) the whole robot workspace; 40,000 configurations inside the whole robot workspace; and (**b**) the target workspace; 336 configurations inside the area where the patient’s leg will be located.

After calibration, the accuracy is validated using 336 configurations that are uniformly distributed inside the Cartesian target workspace. The target workspace is intended to correspond to the area where the patient’s leg will be located (Figure 6b). Also, the accuracy after calibration is evaluated by using the 39,900 configurations (denoted by **Ω_w_**), which are the remaining configurations among the initial set **Ω** composed of 40,000 configurations (Figure 6a) uniformly distributed within the whole robot workspace: 100 calibration configurations are selected from **Ω**, through the observability analysis, to be used in the parameter identification process, and 39,900 configurations are used in the validation after calibration.

After achieving the parameter identification process, the identified parameters are used in the robot kinematics, instead of the nominal parameter values. The next step consists to evaluate the robot accuracy for all validation configurations (**Ω_w_** and **Ω_t_**), by using the following algorithm:

***Loop 1***

For each validation set, **Ω_w_** and **Ω_t_**:

***Loop 2***

For each validation configuration:

(a) Calculate the desired position, by using the identified parameter values and the active joint angles **ψ** = [*q*_1_, *q*_2_,…, *q*_6_]^T^ of the validation configuration. The end-effector position is the translation vector of the homogeneous matrix presented in Equation (1).
(b) Calculate the actual position, by using the actual parameter values (generated by simulation) and the active joint angles of the calibration validation. In case of experimental tests, the actual position is obtained by measurement.
(c) Calculate *x*, *y*, and *z* position errors (*Ex*, *Ey*, and *Ez*), by evaluating the difference between the desired and the actual position, obtained in steps (a) and (b), respectively.
(d) Calculate the composed error ($\sqrt{Ex^{2}+Ey^{2}+Ez^{2}}$) by using results obtained in step (c).

***End Loop 2***

Calculate the mean, the maximum and the standard deviation of all composed errors obtained in *Loop* 1.

***End Loop 1***

The force and torque validation is achieved by using the same algorithm as for position. The only difference is using Equation (17) instead of Equation (1), in step (a).

## 5. Simulation Study

The efficiency of our calibration process is evaluated through a simulation. This process also evaluates the sensitivity of our identification process to the measurement noise, and verifies the effectiveness of the observability analysis for choosing the calibration configurations. Finally, the calibration results (*i.e.*, position accuracy) that were obtained from each of the five observability indices are compared, and the index that gives the best accuracy is used in the actual calibration.

For simulation purposes, the actual parameters’ values are simulated by introducing randomly-generated errors of ±2 mm for the distances, and ±1° for the angles. The differences between the nominal and the actual parameter errors simulate the behavior of a robot with poor accuracy. By using the calibration process, the identified parameters will be as close as possible to their actual values, despite the presence of the measurement errors. The measurement errors that were used in our simulation are ± 1 N and ± 0.2 N·m, for the force and torque, respectively. These errors are an exaggeration of the accuracy of the robot’s force-torque sensor (a Mini 40 from ATI), the details of which are provided by its manufacturer, through a calibration certificate. From this information, force accuracy according to *x*, *y*, and *z* axes was ± 0.25 N, ± 0.2 N, and ± 0.45 N, respectively. The torque accuracy was ± 0.0125 N.m, ± 0.0125 N.m, and ± 0.02 N.m, for x, *y*, and *z* axes.

The measurement errors are generated according to a normal distribution, for each axis (*i.e.*, errors for *F_x_* are generated within ± 1 N, and similarly for *F_y_* and *F_z_*). The data acquisition is simulated by generating 100 measurements (*i.e.*, force and torque errors) for each calibration configuration of the robot. As it is known that the number of identifiable parameters is 29, the number of calibration configurations that are used in the identification process is 100, in order to over-constrain the calibration model.

The measured wrench vector (composed of force and torque) is simulated for each calibration configuration, by substituting the corresponding active joint angles and the actual parameter values (Table 1) in Equation (17). A vector of measurement noise is then added to the obtained wrench vector.

Once all force-torque vectors are generated, the robot parameters are identified as explained in Section 4.2. The identified parameter values are presented in Table 1.

**Figure 7:**
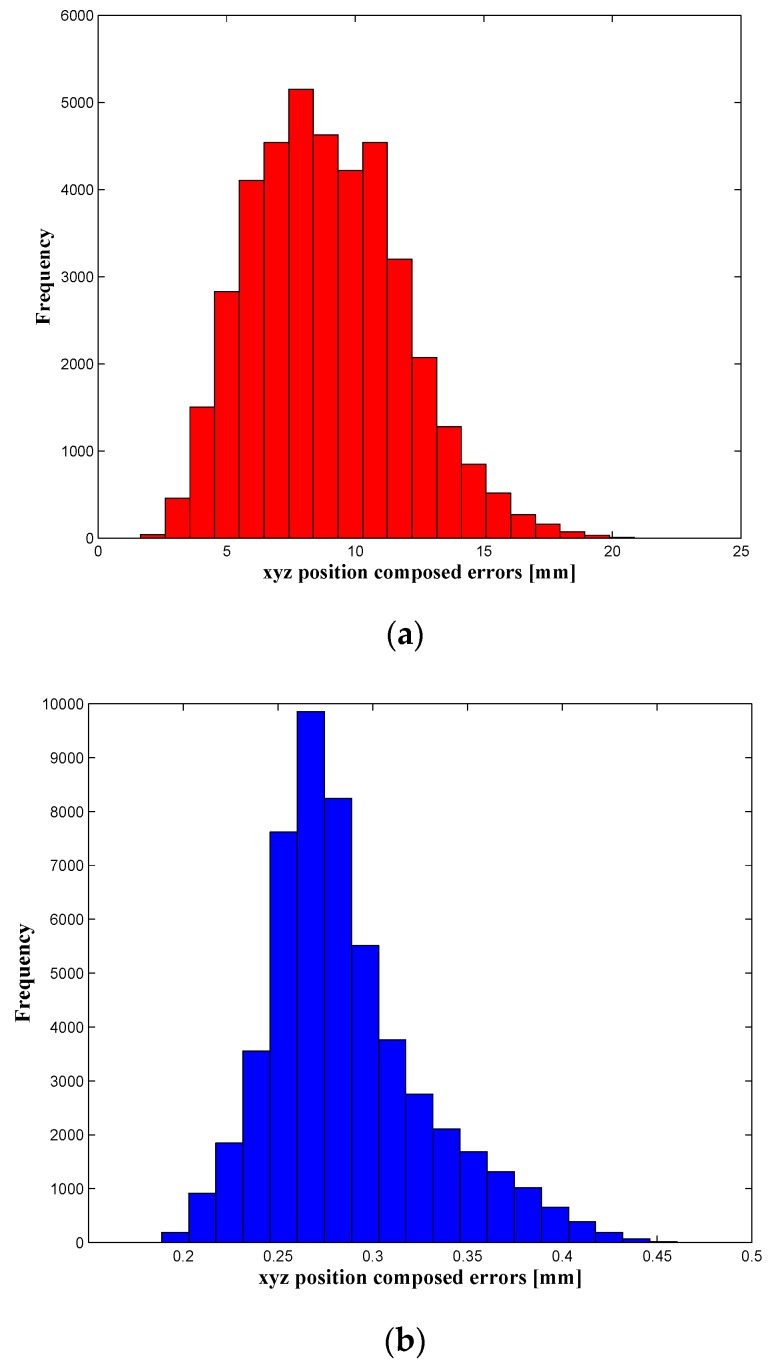
Position errors in the whole workspace (**a**) before and (**b**) after calibration.

**Figure 8:**
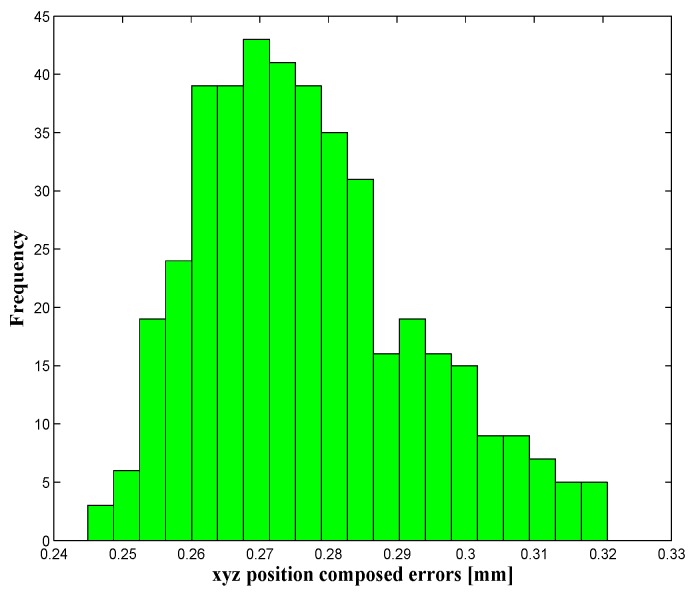
Position errors in the target workspace after calibration.

Once the parameters have been identified, the calibration process is validated. This validation is carried out, as explained in Section 4.3, on two levels: the robot’s position accuracy is assessed in both the whole robot workspace (39,900 configurations) and by using the 336 positions that are uniformly distributed within the target workspace. The force, torque and position errors are summarized in Table 2 and Table 3, and it shows that the position accuracy was improved from 8.9135 mm before calibration to 286 µm, after calibration, inside the target workspace. The wrench errors (Table 4) were highly improved (better than the position accuracy improvement) because in our identification process, only the residuals of force and torque were minimized in the objective function Equation (26). The distribution of the robot’s position errors (before and after calibration) is presented in Figure 7 and Figure 8, which represent the number of occurrences (frequency) of robot *xyz* composed position errors that lie within the ranges of error, presented on the horizontal axis.

A deep analysis of the position errors, for the whole workspace, shows that 0.33% of the evaluated positions have an accuracy lower than 0.2024 mm (mean − 2 × STD), 94.37% are within the range mean ± 2 × STD, and only 5.3% of positions present the poorer accuracy, which is higher than 0.3696 (mean + 2 × STD). The same analysis was achieved for the target workspace, and it shows that 93.0952% of positions have an accuracy within 0.2457 mm and 0.3085 mm (mean ± 2 × STD), and only 6.9048% of positions have errors higher than 0.3085 mm (mean + 2 × STD).

For illustrative purpose, Table 4 shows the accuracy obtained by using each observability index, separately, in the calibration process. Results confirm that *O*_1_ is the most appropriate index for calibrating our robot, since it gives the smallest position (and force/torque) errors, after calibration. Moreover, deeper statistical analyses were carried out on the results obtained by the five indices. First we verified whether the data distributions are Gaussian or not, and then used parametric or non-parametric tests accordingly. Therefore a Kolmogorov-Smirnov test (not shown) was achieved, and it showed that all observability indices provide Gaussian distribution. Based on these results, we decided to use parametric analyses (*i.e.*, ANOVA analysis, and *t*-*test*).

Initially, an ANOVA analysis, with a probability threshold α = 0.05, is used to confirm the objectivity of comparing the five indices (*i.e.*, confirm that there is actually differences between the use of the five indices). Results show that the *F value* is significantly higher than the *F criteria*, which leads to reject the null hypothesis, and therefore conclude that the comparison of results (position accuracy) obtained by using the five indices is meaningful (*i.e.*, results are different, and some indices are better than others).

The ANOVA does not tell where the difference between indices lies. Therefore, an additional test is considered (*t-test*). The *t-test* is used to compare each pair of indices. However, the position errors obtained by using *O*_3_ and *O*_4_ were clearly poorer than results obtained by the other indices (*O*_1_, *O*_2_, and *O*_5_). Thus, only *O*_1_, *O*_2_, and *O*_5_ are considered in the *t-Test*. The results of this test are shown in Table 5, and they show that the position errors obtained by these three indices are quite different, since the *t Stat* value is significantly lower than –*t_Critical_two-tail*, for all pairs of comparisons. Furthermore, to take into account multiple comparison effects, a post-hoc correction is included (Bonferroni correction). As summarized in Table 5, results show that all the *t*-tests were statistically significant (*i.e.*, there are significant differences between the performances of the observability indices).

Based on the aboves tests, we conclude that the fives observability indices allow different results, regarding the robot accuracy after calibration. The analysis of the mean and maximum errors (Table 4) shows that the index *O*_1_ leads to the best robot accuracy: it gives not only the smallest mean errors, but also the smallest maximum errors. Also, *O*_1_ has a small standard deviation, which means that position errors are closely distributed around the mean value.

We recall that in our simulation the used measurement noise was ±1 N. This range of error is an exaggerated error of our wrench sensor (Mini 40, from *API*). For illustrative propose, we achieved other simulations by considering lower measurement noise (Table 6). Results demonstrate that the accuracy after calibration is much better in case of low measurement errors. However, the impact of these errors can be reduced by:

- Using continuous tracking approach: taking several measurements for each robot calibration configuration (*i.e.*, the same applied force), and then averaging the collected data. Most sensors, and data collection card allow a frequency upper than 100 Hz.
- Calibrating the wrench sensor only in a limited range, in which the sensor will be actually used. This will reduce the measurement uncertainty.

## 6. Conclusions

We have presented a self-kinematic calibration approach using a force-torque sensor. With this approach, the position accuracy of a 6-DOF medical robot was significantly improved. The robustness of our calibration model, regarding measurement noise, was confirmed through a simulation study, which also allowed us to conduct an observability analysis in order to identify the most appropriate calibration configurations. The simulation demonstrated that the robot’s position errors were reduced from 12 mm to 0.320 mm for the maximum values, and from 9 mm to 0.277 mm for the mean errors. The calibration method presented in this paper will be tested experimentally in further work.

## Figures and Tables

**Table 1:** Results of the simulated parameter identification.

Param.	Nom.	Actual	Identified, without Meas. Errors	Identified, with Meas. Errors
*A*_1*y*_ (mm)	−233.000	−233.000	−233.000	−233.000
*A*_1*z*_ (mm)	178.000	178.612	178.612	178.659
*C*_1*y*_ (mm)	−83.000	−84.005	−84.005	−84.002
*C*_1*z*_ (mm)	438.000	438.000	438.000	438.000
*A*_2*y*_ (mm)	−233.000	−232.267	−232.267	−232.294
*A*_2*z*_ (mm)	178.000	177.325	177.325	177.485
*C*_2*y*_ (mm) *	−83.000	−83.000	−83.000	−83.000
*C*_2*z*_ (mm)	438.000	438.112	438.112	438.210
*L*_11_ (mm)	400.000	400.730	400.730	400.742
*L*_12_ (mm)	520.000	520.221	520.221	520.321
*L*_13_ (mm)	400.000	398.485	398.485	398.502
*L*_14_ (mm)	520.000	520.332	520.332	520.271
*L*_21_ (mm)	400.000	399.799	399.799	399.774
*L*_22_ (mm)	520.000	518.336	518.336	518.505
*L*_23_ (mm) *	400.000	400.000	400.000	400.000
*L*_24_ (mm)	520.000	520.445	520.445	520.460
*d*_41_ (mm)	41.500	41.660	41.660	41.657
*d*_42_ (mm) *	41.500	41.500	41.500	41.500
*d*_5_ (mm) *	0.000	0.000	0.000	0.000
*x*_T_** (mm) *	0.000	0.000	0.000	0.000
*y*_T_** (mm) *	0.000	0.000	0.000	0.000
*z*_T_** (mm) *	134.600	134.600	134.600	134.600
*α*_T_ (º) *	67.512	67.512	67.512	67.512
*β*_T_ (º) *	0.000	0.000	0.000	0.000
*γ*_T_ (º) *	0.000	0.000	0.000	0.000
*x*_0_** (mm) *	109.000	109.000	109.000	109.000
*y*_0_** (mm) *	139.000	139.000	139.000	139.000
*z*_0_ (mm) *	−31.000	−31.000	−31.000	−31.000
*α*_0_ (º) *	0.000	0.000	0.000	0.000
*β*_0_ (º)	0.000	0.525	0.525	0.531
*γ*_0_ (º)	0.000	−0.134	−0.134	−0.176
*δq*_2_ (º)	0.000	0.313	0.313	0.342
*δq*_3_ (º)	0.000	−0.052	−0.052	−0.014
*δq*_4_ (º)	0.000	0.500	0.500	0.529
*δq*_5_ (º)	0.000	0.135	0.135	0.173
*δq*_6_ (º)	0.000	0.102	0.102	0.097
*x*_S_ (mm) *	41.500	41.500	41.500	41.500
*y*_S_ (mm) *	0.000	0.000	0.000	0.000
*z*_S_ (mm) *	41.700	41.700	41.700	41.700
*α*_S_ (º)	−67.512	−67.397	−67.397	−67.430
*β*_S_ (º)	0.000	−0.525	−0.525	−0.528
*γ*_S_ (º)	0.000	0.185	0.185	0.196
*x*_G_ (mm)	0.000	0.576	0.576	0.571
*y*_G_ (mm)	0.000	0.059	0.059	0.042
*z*_G_ (mm)	152.400	153.132	153.132	153.136
*m*_Tool_ (g)	0.365	0.365	0.365	0.365

**Table 2:** Composite force and torque errors before and after calibration.

	Force (N)				Torque (N.m)			
	Mean	Mean % w.r.t. Max	Max	STD	Mean	Mean % w.r.t. Max	Max	STD
**Whole Workspace**								
**Before**	0.0703	24.84	0.2830	0.0454	0.0100	28.33	0.0353	0.0056
**After**	0.0004	20.00	0.0020	0.0003	0.0000	0.00	0.0002	0.0000
**Improvement %**	99.43	-	99.29	99.34	100.00	-	99.43	100.00
**Target Workspace**								
**Before**	0.0474	46.33	0.1023	0.0264	0.0055	60.44	0.0091	0.0018
**After**	0.0008	61.54	0.0013	0.0002	0.0002	66.67	0.0003	0.0000
**Improvement %**	98.31	-	98.73	99.24	96.36	-	96.70	100.00

**Table 3:** Composite position errors before and after calibration.

	Mean (mm)	Mean% w.r.t. Max	Max (mm)	STD (mm)
****	**Whole Workspace**			
**Before**	8.9135	42.76	20.8437	2.9138
**After**	0.2860	60.24	0.4748	0.0418
**Improvement %**	96.79	-	97.72	98.57
****	**Target Workspace**			
**Before**	9.0118	73.64	12.2382	1.6537
**After**	0.2771	86.43	0.3206	0.0157
**Improvement %**	96.93	-	97.38	99.05

**Table 4:** Composite force, torque and position errors after calibration using the five observability indices.

	Force (N)			Torque (N.m)			Position (mm)		
	Mean	Max	STD	Mean	Max	STD	Mean	Max	STD
**Whole Workspace**									
***O*_1_**	0.0004	0.0020	0.0003	0.0000	0.0002	0.0000	0.2860	0.4748	0.0418
***O*_2_**	0.0024	0.0189	0.0031	0.0003	0.0021	0.0003	0.3818	0.9391	0.1070
***O*_3_**	0.0644	0.0497	0.1011	0.0114	0.0553	0.0112	8.8182	10.1284	0.4050
***O*_4_**	0.0122	0.0356	0.0099	0.0013	0.0040	0.0011	4.5199	6.2311	0.3134
***O*_5_**	0.0006	0.0031	0.0004	0.0001	0.0003	0.0000	0.4552	0.6767	0.0637
**Target Workspace**									
***O*_1_**	0.0005	0.0012	0.0002	0.0001	0.0001	0.0000	0.2771	0.3206	0.0157
***O*_2_**	0.0008	0.0014	0.0003	0.0002	0.0003	0.0000	0.3527	0.4493	0.0231
***O*_3_**	0.0387	0.0569	0.0282	0.0025	0.0043	0.0052	7.5427	10.7637	0.7089
***O*_4_**	0.0124	0.0228	0.0047	0.0013	0.0025	0.0005	4.4836	5.8925	0.1638
***O*_5_**	0.0003	0.0008	0.0001	0.0000	0.0001	0.0000	0.4318	0.5937	0.0320

**Table 5:** Results of the *t*-test comparing pairs of *O*_1_, *O*_2_, and *O*_5_.

*t*-Test			
	***O*_1_*vs. O*_2_**	***O*_1_*vs. O*_5_**	***O*_2_*vs. O*_5_**
***t Stat***	−655.874	−304.773	421.3063
***t Critical two-tail***	1.960001	1.959993	1.959993
**Post-hoc correction (Bonferroni correction) *Target p-value =*** $\frac{\alpha }{\text{number}\;\text{of}\;\text{t-Test}}=\frac{0.05}{3}=0.0167$			
***p-value(from t-test)***	0.000000	0.000000	0.000000
***Test statistically significant***	yes	yes	yes

**Table 6:** Composite position errors after calibration, inside the target workspace, using different measurement errors.

Err. (N)	Mean	Max	STD
**±0.2**	0.0759	0.0885	0.0039
**±0.4**	0.1124	0.1992	0.0042
**±0.6**	0.1874	0.2845	0.0070
**±0.8**	0.2174	0.3014	0.0103
**±1**	0.2771	0.3206	0.0157
